# Computational investigation of spin-resolved energy landscapes of FeC_4_H_2_^+^ and their astrochemical implications

**DOI:** 10.3389/fchem.2026.1794369

**Published:** 2026-03-19

**Authors:** Shilpa Shajan, Krishnan Thirumoorthy

**Affiliations:** 1 Department of Chemistry, School of Advanced Sciences, Vellore Institute of Technology, Vellore, India; 2 School of Computer Science and Engineering, Vellore Institute of Technology, Vellore, India

**Keywords:** astrochemistry, chemical bonding, interstellar medium, iron-bearing molecules, isomers, missing iron

## Abstract

The enduring enigma of iron depletion in the interstellar medium serves as a compelling impetus for an in-depth exploration of iron-bearing molecular species. The cationic system FeC_4_H_2_^+^ is theoretically explored in the doublet, quartet, and sextet electronic states using density functional theory. The geometry corresponding to the least energy within the quartet state represents the global minimum, thus substantiating the assertion that the quartet constitutes the ground state of this particular system. The bonding characterization of the three lowest-energy geometries across the three electronic states is explored using quantum-chemical tools, including Wiberg bond indices, natural atomic charges, and the electron localization function. The recent detection of the FeC radical in the interstellar medium implies that the investigated systems are strong candidates for radioastronomical observation and contribute to the body of knowledge on iron chemistry in the cosmos.

## Introduction

The deepening collaboration of astrochemistry and quantum science has revolutionized our understanding of molecular detection, formation, and endurance in the vast realm of interstellar chemistry over the past decades. Nearly a century has elapsed since astronomers first identified the elusive methylidyne radical traversing the interstellar medium (ISM) in 1937, a groundbreaking moment in our understanding of the cosmic chemistry that connects the universe (Swings and Rosenfeld, 1937). Currently, that solitary discovery has evolved into a molecular myriad of over 340 molecules in the interstellar medium or circumstellar shells, from simple diatomic radicals to intricate organics (Müller et al., 2005). Among these, the ionic species present at relatively low abundance are ubiquitous and significantly influence the governing chemical evolution across the cosmos (Larsson et al., 2012; McGuire et al., 2020). Cations in the ISM arise from diverse ionization processes, including ultraviolet (UV) and X-ray photons from nearby stars, cosmic rays, shock waves, high-energy particles ejected by stellar winds or supernovae, and ion-neutral reactions that propagate charge transfer in the gas phase. One of the early detections of interstellar molecules includes the CH^+^ (McKellar, 1940), and subsequent discoveries have expanded the molecular inventory to complex species such as the Buckminster-Fullerene cation, which has been conclusively identified as the diffuse interstellar band (DIB) carrier (Campbell et al., 2015; Cordiner et al., 2019). These discoveries ignited a surge of research into the convoluted ion chemistry in the cosmos.

Paradoxically, its cosmic abundance as a refractory element, iron, is conspicuously missing in the ISM. Iron is produced predominantly in massive stars through core-collapse supernovae and in Type Ia supernova explosions (Dwek, 2016). In a quest to decode the enigma of missing iron, many reservoirs have been proposed, which include the iron-bearing nanoparticles, complexation of iron with polycyclic aromatic hydrocarbons (PAH), metallic dust grains, iron pseudocarbynes, and iron-doped cosmic nano silicates (Banerjee et al., 2025; Bilalbegović et al., 2017; Jensen and Snow, 2007; Jones, 1990; McDonald et al., 2010; Tarakeshwar et al., 2019). As of now, only two iron-bearing molecules have been identified in ISM: FeCN, detected in the circumstellar shell of IRC+10216, and the recent detection of the FeC radical (Koelemay and Ziurys, 2023; Zack et al., 2011). The detection of the FeC radical has provided strong motivation to investigate iron-carbon molecular systems. In 2013, Chandra and co-workers investigated small iron-bearing ring molecules of astrophysical relevance, encompassing the FeC_2,3_ and FeC_n_H_m_ (n = 2,3 & m = 2,4) systems (Chang et al., 2013).

The detection of other metal-containing molecules in the interstellar medium, notably SiC_x_ (x = 1–4) (Cernicharo et al., 1989; Ohishi et al., 1989; Apponi et al., 1999; Massalkhi et al., 2023) and a range of magnesium-bearing molecules including MgCN (Ziurys et al., 1995), MgC_3_N (Cernicharo et al., 2019), MgC_5_N (Pardo et al., 2021), MgC_4_H (Cernicharo et al., 2019), and MgC_6_H (Pardo et al., 2021), has provided strong impetus for exploring iron-carbon-hydrogen systems. The presence of exotic cumulene carbenes (C_*n*_H_2_, n = 3,4,5) in the interstellar medium suggests a possible role as precursor species in the chemistry leading to iron-carbon hydrides (Apponi et al., 2000; Cabezas et al., 2021; Kawaguchi et al., 1991; Langer et al., 1997). Our group has previously explored the neutral and dicationic forms of FeC_4_H_2_, and in this work, we focus on FeC_4_H_2_^+^ as a potential reservoir of the missing iron in the ISM (Shajan et al., 2024; Shajan and Thirumoorthy, 2024). The recent discovery of MgC_4_H^+^, MgC_6_H^+^, MgC_3_N^+^, and MgC_5_N^+^ by Cernicharo has spurred renewed interest in cationic iron-bearing species (Cernicharo et al., 2023). The spectral characteristics of Fe^+^(H_2_O) analyzed by photodissociation spectroscopy have suggested that smaller iron-containing molecular entities are promising candidates as carriers of DIBs (Juanes et al., 2024). Numerous investigations have been conducted on iron-carbon hydrides, both theoretically and experimentally, which emphasized the significance of iron, a unique element due to its position between the early and late transition metals (Nash et al., 1996; Romanescu et al., 2012; Ryzhkov and Delley, 2012; Zhu and Li, 2009). The theoretical exploration of FeCH_n_^0/+^(n = 1,2,3) by Frenking and co-workers laid the foundation for subsequent experimental and theoretical studies (Veldkamp and Frenking, 1992). Mass-selected photodetachment photoelectron spectroscopy of small iron-carbon-hydrogen molecules has led to the identification of FeC_4_H_2_ (Drechsler and Boesl, 2003); however, to the best of our knowledge, no subsequent studies have been reported.

The aforementioned discourse indicates that the investigation of cationic iron-bearing molecules remains insufficiently addressed, thereby contributing to understanding the elusive iron conundrum and potentially uncovering solutions to additional enigmas within the ISM, such as DIBs. The synthesis and laboratory spectroscopic investigation of small ions and radicals pose significant experimental challenges, underscoring the essential role of computational modeling. Quantum chemistry serves as a fundamental pillar in the domain of astrochemical investigation, facilitating computational analyses that yield accurate molecular configurations and spectroscopic information, while simultaneously providing essential characteristics to support comprehensive astrochemical modeling (Fortenberry, 2024).

In the present work, the potential energy surfaces (PES) of FeC_4_H_2_^+^ are comprehensively explored in the doublet, quartet, and sextet electronic states, along with bonding analyses of the lowest energy geometries. Although detailed energetic investigation offering spectroscopic insights remains scarce for this cationic species, the current investigation fills a vital void. This investigation delivers crucial data to enhance our grasp of iron chemistry in the ISM, potentially revealing reservoirs of the elusive missing iron.

## Computational methodology

All the quantum chemical calculations in this work were carried out using the Gaussian 16 package (Frisch, et al., 2016). Structural optimizations and harmonic frequency analyses for characterizing stationary points were performed using density functional theory (DFT) with the U*ω*B97X-D functional, a range-separated hybrid approach enhanced by Grimme’s D2 dispersion correction (Chai and Head-Gordon, 2008). The Stuttgart/Dresden Effective core potential (ECPs) incorporating the SDD basis set was used for the heavy element iron (Curtiss et al., 1995; Dolg et al., 1986), and 6–311++G(2D,2P) was utilized for the carbon and hydrogen atoms (Fuentealba et al., 1982). The exploration of the potential energy surfaces was performed primarily based on chemical intuition to construct plausible initial geometries, while an in-house Python script was subsequently employed for systematic structure generation (Shajan and Thirumoorthy, 2024; Thimmakondu et al., 2022). Wiberg bond indices (WBI) and natural atomic charges (NAC) were obtained using the natural bond orbital (NBO) (Reed et al., 1985) methodology as implemented in Gaussian 16 (Frisch et al., 2016). The electron localization function (ELF) was performed using the wavefunction file generated using Gaussian with the Multiwfn program (Lu and Chen, 2012).

## Results and discussions

**FIGURE 1:**
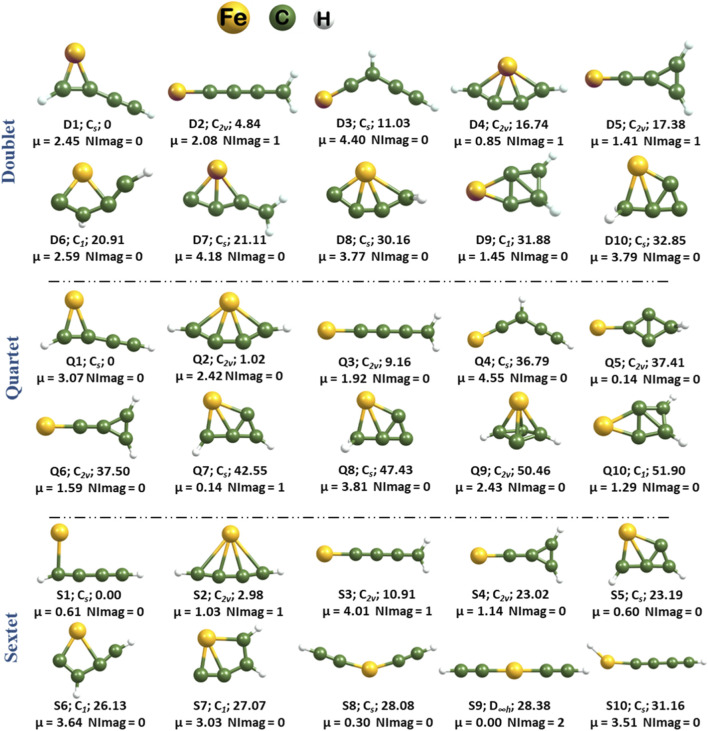
Ten low-lying isomers of FeC_4_H_2_^+^ in their doublet, quartet, and sextet states. The point group, zero-point-corrected relative energies (kcal mol^-1^), dipole moment (in Debye), and NImag calculated at the U*ω*B97X-D/SDD & 6–311++G(2d,2p) level. The other isomers identified on the potential energy surfaces in the doublet, quartet, and sextet states are provided in the Supplementary Material.

Exploration of the potential energy surfaces of FeC_4_H_2_^+^ revealed 39 distinct isomers in the doublet state and 26 isomers each in the quartet and sextet states at the U*ω*B97X-D/SDD & 6–311++G(2d,2p) level of theory. The first ten low-lying isomers of the FeC_4_H_2_^+^ system in the doublet, quartet, and sextet states, detailing their point group, zero-point vibrational energy (ZPVE) corrected relative energies, dipole moment (in Debye), and the number of imaginary frequencies (NImag), are presented in Figure 1. The remaining optimized geometries are included in the Supporting Information (Supplementary Figures S1–S3). The comparative analysis of the doublet, quartet, and sextet electronic states reveals that the quartet state is the ground state of this cationic system.

The selected level of theory has been widely applied to iron-containing systems and is known to provide reliable equilibrium geometries, spin-state energetics, and relative stability trends within the typical DFT uncertainty range for 3d transition metals. The UωB97X-D functional was chosen for its long-range exact exchange and empirical dispersion corrections, which enable a balanced treatment of electron correlation relevant to transition-metal-carbon systems. Range-separated hybrids such as ωB97X-D have demonstrated consistent performance for iron complexes, particularly for properties sensitive to correlation effects. The SDD effective core potential was employed for Fe to account for scalar relativistic effects while maintaining computational efficiency. For the remaining atoms, the 6–311++G(2d,2p) basis set was used to provide a balanced valence description with polarization and diffuse functions, the latter being important for accurately describing open-shell cationic species, including charge delocalization and spin density distribution (Aktürk and Sebetci, 2016; Flores-Leonar et al., 2017; Johnson et al., 2025; Listyarini et al., 2019; Minenkov et al., 2012; Murat et al., 2010).

In the doublet electronic state, analysis of the 39 isomers reveals that nine correspond to transition-state geometries, four to higher-order saddle points, and the remaining structures are confirmed as local minima. For the quartet and sextet states, each featuring 26 isomers, five transition-state geometries are identified in the PES of both. Furthermore, the quartet state features four higher-order saddle points, whereas six are found in the sextet state. In all examined electronic states, the lowest-energy isomers adopt an identical structural motif, with only subtle geometric variations. Among these isomers, the quartet isomer **Q1** is energetically the most favorable and corresponds to the global minimum on the potential energy surface of FeC_4_H_2_^+^. Almost all initial low-lying isomers are observed across the three electronic states, albeit with shifts in their relative energies. The **D4**, **Q2**, and **S2** represent planar tetracoordinate iron (ptFe) geometries previously characterized in our earlier work on dicationic and neutral forms of FeC_4_H_2_, and their presence is reaffirmed on the potential energy surfaces of this cationic system (Shajan et al., 2024; Shajan and Thirumoorthy, 2024). The ptFe geometries are minima in the ground electronic state, while in other states they are transition-state structures. A closer examination of the isomers shown in Figure 1 indicates that these geometrical configurations could plausibly result from interactions between iron atoms and the cumulene carbenes identified in the ISM. The positive dipole moments exhibited by all low-lying geometries bolster their potential for detection in the ISM. In particular, isomers across the three electronic states with dipole moments exceeding 2 Debye stand out as highly promising targets for astronomical identification, according to Ellinger and coworkers (Ellinger et al., 2020).

Spectroscopic parameters provide essential evidence for molecular identification in laboratory experiments, facilitating their subsequent detection in the ISM. Notably, microwave spectroscopy necessitates a non-zero permanent dipole moment, as the intensity of spectral lines scales proportionally to its square. Table 1 summarizes the inertial-axis dipole moments (with Cartesian components), rotational constants, and centrifugal distortion constants for the ten lowest-energy isomers of the quartet ground state. Subtle geometric perturbations exert a profound influence on spectroscopic parameters. Thus, precise elucidation of molecular structures proves indispensable for pinpointing candidate molecules and their prevailing conformers. Every isomer displays a non-zero dipole moment, with five isomers exceeding 2 Debye, making them strong contenders for astronomical detection. Although this parameter plays a significant role in molecular observability (Ellinger et al., 2020), it represents only one of several contributing factors. The molecule’s thermodynamic stability provides additional support for its interstellar viability. Despite the absence of direct experimental data for comparative analysis, our calculated rotational constants show correlation with those obtained from empirical studies of FeC_4_ and theoretical estimates of iron-bearing molecules in astronomical contexts (Chang et al., 2013; Steglich et al., 2014). For the identified FeC_4_ isomer, which is a structure similar to our ptFe (**Q2**), the calculated rotational constants are: *A* = 7584.75 MHz, *B* = 6055.81 MHz, and *C* = 3387.65 MHz. Our computed rotational constants for **Q2** align closely with these values, underscoring significant structural parallels, likely a shared planar tetracoordinate iron framework with comparable Fe-C and C-C bonding patterns, despite the difference in charge and protonation. Such correspondence supports the structural assignment of our ptFe-like FeC_4_ isomer and could guide future high-resolution rotational spectroscopy in the laboratory or targeted astronomical searches for iron-bearing carbon species in the ISM, where they may help resolve the “missing iron” problem. The centrifugal distortion constants determined for these initial low-lying geometries offer useful benchmarks for experimentalists, aiding in the identification and characterization of these species through rotational spectroscopy.

**TABLE 1:** Inertial axis dipole moment components, absolute dipole moments (Debye), rotational constants (MHz), and centrifugal distortion constants of the initial ten low-lying isomers of FeC_4_H_2_^+^ in their quartet state.

Isomer	μ_a_	μ_b_	μ_c_	|μ|	A_e_	B_e_	C_e_	D_J_	D_K_	D_JK_	d_1_	d_2_
Q1	−2.43	−1.87	0.00	3.07	10182.346	2159.601	1781.7125	16.28x10^−4^	11.94x10^−2^	−17.20x10^−3^	46.63x10^−5^	60.42x10^−4^
Q2	0.00	0.00	2.42	2.42	6665.666	4883.647	2818.588	73.56x10^−4^	−16.03x10^−3^	95.04x10^−4^	28.82x10^−4^	31.91x10^−3^
Q3	0.00	0.00	1.92	1.92	287523.839	1008.799	1005.272	29.38x10^−6^	23.36x10^−2^	13.91x10^−3^	−10.55x10^−8^	−74.66x10^−4^
Q4	2.20	3.11	0.00	3.81	8632.056	3791.657	2681.582	10.68x10^−4^	26.13x10^−4^	13.35x10^−4^	−33.44x10^−5^	−27.70x10^−4^
Q5	0.00	0.00	0.14	0.14	33358.976	1591.824	1535.017	14.81x10^−5^	13.47x10^−2^	52.99x10^−4^	−57.41x10^−7^	−31.28x10^−4^
Q6	0.00	0.00	1.59	1.59	33154.674	1333.522	1281.960	68.37x10^−6^	62.84x10^−3^	63.85x10^−3^	−28.59x10^−7^	−36.14x10^−4^
Q7	0.07	0.12	0.00	0.14	10215.286	3457.417	2583.140	85.56x10^−5^	74.22x10^−4^	−40.00x10^−5^	−24.72x10^−5^	−22x29x10^−4^
Q8	2.20	3.11	0.00	3.81	8632.056	3791.657	2681.582	10.68x10^−4^	26.13x10^−4^	13.35x10^−4^	−33.44x10^−5^	−27.70x10^−4^
Q9	0.00	0.00	2.43	2.43	8791.637	4782.928	3840.194	17.19x10^−4^	−75.98x10^−4^	78.17x10^−4^	−34.77x10^−5^	−13.75x10^−4^
Q10	1.10	−0.29	0.60	1.29	16903.566	2566.430	2283.542	58.25x10^−5^	66.25x10^−3^	−20.82x10^−4^	91.89x10^−6^	37.56x10^−4^

**FIGURE 2:**
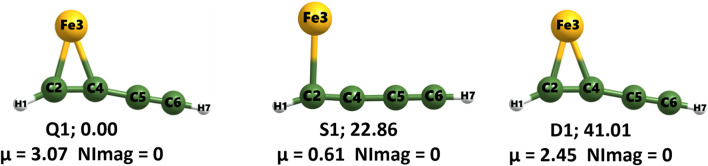
The three lowest-energy geometries in their quartet, sextet, and doublet states, along with their relative energies (in kcal mol^-1^) with respect to the global minimum Q1.

Figure 2 presents the three lowest-energy geometries in the quartet, sextet, and doublet states, with their relative energy with respect to the global minimum **Q1**. Despite sharing the same structural framework, this molecular geometry varies markedly across the three electronic states, highlighting the strong influence of spin multiplicity. Such behavior is characteristic of transition-metal complexes and arises from changes in d-orbital occupation and metal-ligand bonding interactions. Relative to the global minimum **Q1**, the **S1** and **D1** geometries are energetically less favorable by 22.86 and 41.01 kcal mol^-1^, respectively, highlighting a pronounced stabilization of the quartet ground state. Apart from **S1**, both low-energy structures possess dipole moments exceeding 2 Debye, thereby enhancing their detectability in the ISM.

Table 2 presents the geometrical parameters (bond lengths and bond angles) and WBI values for the **Q1**, **S1**, and **D1** geometries. Across the three geometries, the two Fe–C bond lengths range from 1.82 to 2.83 Å, suggesting moderate to weak single-bond interactions between iron and carbon. **S1** exhibits significantly elongated Fe–C bonds, and one of them is missing compared to the other two states, indicating substantial structural reorganization driven by differences in spin multiplicity. The C2–C4 bond distances progressively decrease across the **D1**, **Q1**, and **S1** states, indicating a partial double bond to triple bond character. In contrast, the C5–C6 bond lengths remain unchanged in all three geometries, consistent with a triple bond character.

**TABLE 2:** Geometrical parameters (Å and degrees) and Wiberg bond indices of **Q1**, **S1**, and **D1**.

Geometrical parameters	Q1		S1		D1	
	Bond lengths	WBI	Bond lengths	WBI	Bond lengths	WBI
R(H1,C2)	1.07	0.88	1.08	0.87	1.08	0.88
R(C2,Fe3)	2	0.37	2.23	0.24	1.82	0.82
R(Fe3,C4)	2.05	0.32	2.83	0.03	1.86	0.70
R(C2,C4)	1.24	2.39	1.23	2.45	1.29	2.02
R(C4,C5)	1.38	1.18	1.35	1.26	1.38	1.19
R(C5,C6)	1.2	2.7	1.2	2.63	1.2	2.70
R(C6,H7)	1.07	0.92	1.07	0.92	1.07	0.92
θ(H1,C2,Fe3)	129.10		107.74		139.92	
θ(C2,Fe3,C4)	35.71		24.76		40.87	
θ(Fe3,C4,C5)	121.31		123.79		135.14	
θ(C4,C5,C6)	178.13		179.45		178.14	
θ(C5,C6,H7)	178.73		179.61		178.87	

WBI provides insights into the covalent character of the bonding in the isomers. WBI values of iron-carbon suggest that significant covalent characteristics of the Fe–C interactions in the three geometries, whereas reduced covalent character is observed for Fe3–C4 in **S1**. Strong covalent character is indicated by WBI for the carbon chain, which extends from the ring to the periphery across the three structures. Turning to the bond angles of the three geometries, the **S1** exhibits pronounced deviations compared to **Q1** and **D1**. In particular, the θ(C2, Fe3, C4) bond angle is significantly smaller in **S1**, indicating a pronounced tilting of the molecular framework relative to the other two geometries. An increase in the number of unpaired electrons leads to pronounced geometric distortion. However, this higher-spin electronic state is energetically favored over the corresponding lower-spin state.

**FIGURE 3:**
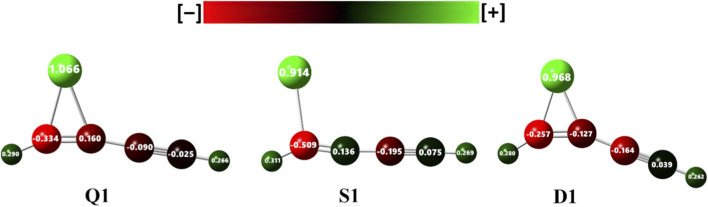
The natural atomic charges (|e|) on all atoms of Q1, S1, and D1.

For the charge analysis, we employed natural atomic charges from NBO, a well-established method for analyzing molecular charge distributions. Figure 3 shows the NAC on all the atoms of the geometries **Q1**, **S1**, and **D1**. Among the three geometries, the iron atom in the lowest-energy **Q1** exhibits a large positive NAC, indicating enhanced charge transfer to the carbon chain relative to the other two. The values are 1.066, 0.914, and 0.968 for the **Q1**, **S1**, and **D1**, respectively. The distribution of negative to mildly positive charges on the carbon chain supports significant charge transfer from the iron atom to the carbon framework. Analysis of **S1** reveals a pronounced partial negative charge on C2 compared to the other geometries, which plays a crucial role in the geometrical deformations, as the localized electron density modifies electrostatic interactions, thereby stabilizing the structure. The significant charge difference between iron and the carbon chain indicates the robust electrostatic attraction in the three geometries.

**FIGURE 4:**
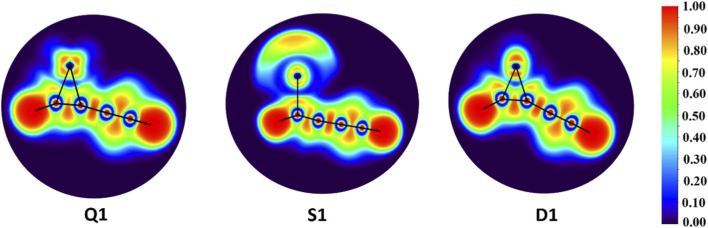
Color-filled map of ELF of Q1, S1, and D1.

The topological examination, specifically the electron localization function (ELF), serves as a methodological tool for assessing the probability of electron pairing, represented as a three-dimensional real-space function. It also provides significant insight into the electronic structure and the classification of chemical bonds within a molecular entity. A larger ELF value suggests the presence of localized electrons and a reduced tendency for delocalization, while lower ELF values denote a diminished degree of electron localization or covalent interactions. Figure 4 illustrates the color-filled maps of ELF of **Q1**, **S1**, and **D1**. The hues ranging from orange to yellow indicate a reduction in localization, consistent with covalent bonding of the carbon chain across the three geometrical configurations. In **S1**, the elevated number of unpaired electrons results in a larger electron density concentrated on the iron center compared to the other two geometries. This metal-centered electronic distribution rationalizes the absence of an Fe–C bond and correlates well with the reduced natural atomic charge on iron. Enhanced interactions between the carbon chain and iron centers are evident in the **Q1** and **D1** geometries, surpassing those observed in **S1**. In **D1**, electron localization is notably greater than in the other geometries, implying limited delocalization and thereby accounting for its reduced stability.

## Conclusion

In summary, this investigation provides a comprehensive exploration of the potential energy surfaces of the FeC_4_H_2_^+^ system in the doublet, quartet, and sextet electronic states. The **Q1** isomer is demonstrated to exhibit maximal stability, thereby designating the quartet state as the ground state for this system. Furthermore, this **Q1** isomer maintains the lowest energy configuration across all three electronic states. The spectroscopic parameters of the initial ten isomers of ground quartet states are presented to assist experimentalists in the potential laboratory detection of these species, which may subsequently facilitate their identification in the ISM. Bonding characteristics of the three lowest-energy geometries (**Q1**, **S1**, and **D1**) across the three electronic states were examined using quantum-chemical methods, including Wiberg bond indices, natural atomic charges, and electron localization functions. The cationic system explored in this study holds potential to address the longstanding puzzle of missing iron in the ISM, equipping radio astronomers, laboratory spectroscopists, and synthetic chemists with tools for imminent detection of these isomers.

## Data Availability

The original contributions presented in the study are included in the article/Supplementary Material, further inquiries can be directed to the corresponding author.

## References

[B1] Aktürk A. Sebetci A. (2016). BH-DFTB/DFT calculations for iron clusters. AIP Adv. 6, 055103. 10.1063/1.4948752

[B2] Apponi A. J. McCarthy M. C. Gottlieb C. A. Thaddeus P. (1999). Astronomical detection of rhomboidal SiC_3_ . Astrophys. J. 516, L103–L106. 10.1086/311998

[B3] Apponi A. J. McCarthy M. C. Gottlieb C. A. Thaddeus P. (2000). Laboratory detection of four new cumulene carbenes: H_2_C_7_, H_2_C_8_, H_2_C_9,_ and D_2_C_10_ . Astrophys. J. 530, 357–361. 10.1086/308353

[B4] Banerjee D. George L. E. Chatterjee S. Koley D. (2025). *In silico* investigation of Fe-Doped cosmic nanosilicates. ACS Earth Space Chem. 9, 2621–2642. 10.1021/acsearthspacechem.5c00186

[B5] Bilalbegović G. Maksimović A. Mohaček-Grošev V. (2017). Missing Fe: hydrogenated iron nanoparticles. Mon. Not. R. Astron. Soc. Lett. 466, L14–L18. 10.1093/mnrasl/slw226

[B6] Cabezas C. Tercero B. Agúndez M. Marcelino N. Pardo J. R. De Vicente P. (2021). Cumulene carbenes in TMC-1: astronomical discovery of l-H_2_C_5_ . Astron. Astrophys. 650, 1–5. 10.1051/0004-6361/202141274 34334798 PMC7611420

[B7] Campbell E. K. Holz M. Gerlich D. Maier J. P. (2015). Laboratory confirmation of C_60_ ^+^ as the carrier of two diffuse interstellar bands. Nature 523, 322–323. 10.1038/nature14566 26178962

[B8] Cernicharo J. Gottlieb C. A. Guelin M. Thaddeus P. Vrtilek J. M. (1989). Astronomical and laboratory detection of the SiC radical. Astrophys. J. 341, 25–28. 10.1086/185449

[B9] Cernicharo J. Cabezas C. Pardo J. R. Agúndez M. Bermúdez C. Velilla-Prieto L. (2019). Discovery of two new magnesium-bearing species in IRC+10216: MgC_3_N and MgC_4_H. Astron. Astrophys. 630, 1–10. 10.1051/0004-6361/201936372 31579315 PMC6774763

[B10] Cernicharo J. Cabezas C. Pardo J. R. Agúndez M. Roncero O. Tercero B. (2023). The magnesium paradigm in IRC +10216: discovery of MgC_4_H^+^, MgC_3_N^+^, MgC_6_H^+^, and MgC_5_N^+^ . Astron. Astrophys. 672, 1–13. 10.1051/0004-6361/202346467

[B11] Chai J. D. Head-Gordon M. (2008). Long-range corrected hybrid density functionals with damped atom-atom dispersion corrections. Phys. Chem. Chem. Phys. 10, 6615–6620. 10.1039/b810189b 18989472

[B12] Chang C. Patzer A. B. C. Kegel W. H. Chandra S. (2013). Small Fe bearing ring molecules of possible astrophysical interest: molecular properties and rotational spectra. Astrophys. Space Sci. 347, 315–325. 10.1007/s10509-013-1516-0

[B13] Cordiner M. A. Linnartz H. Cox N. L. J. Cami J. Najarro F. Proffitt C. R. (2019). Confirming interstellar C_60_ ^+^ using the hubble space telescope. Astrophys. J. Lett. 875, L28. 10.3847/2041-8213/ab14e5

[B14] Curtiss L. A. McGrath M. P. Blaudeau J. P. Davis N. E. Binning R. C. Radom L. (1995). Extension of Gaussian-2 theory to molecules containing third-row atoms Ga-Kr. J. Chem. Phys. 103, 6104–6113. 10.1063/1.470438

[B15] Dolg M. Wedig U. Stoll H. Preuss H. (1986). Energy-adjusted ab *initio* pseudopotentials for the first row transition elements. J. Chem. Phys. 86, 866–872. 10.1063/1.452288

[B16] Drechsler G. Boesl U. (2003). Mass selective photodetachment photoelectron spectroscopy: small transition metal (Fe, Ni) carbon hydrogen compounds. Int. J. Mass Spectrom. 228, 1067–1082. 10.1016/S1387-3806(03)00220-3

[B17] Dwek E. (2016). Iron: a key element for understanding the origin and evolution of interstellar dust. Astrophys. J. 825, 136. 10.3847/0004-637x/825/2/136 32747835 PMC7398334

[B18] Ellinger Y. Pauzat F. Markovits A. Allaire A. Guillemin J.-C. (2020). The quest of chirality in the interstellar medium. Astron. Astrophys. 633, A49. 10.1051/0004-6361/201936901

[B19] Flores-Leonar M. M. Moreno-Esparza R. Ugalde-Saldívar V. M. Amador-Bedolla C. (2017). Further insights in DFT calculations of redox potential for iron complexes: the ferrocenium/ferrocene system. Comput. Theor. Chem. 1099, 167–173. 10.1016/j.comptc.2016.11.023

[B20] Fortenberry R. C. (2024). Quantum chemistry and astrochemistry: a match made in the heavens. J. Phys. Chem. A 128, 1555–1565. 10.1021/acs.jpca.3c07601 38381079

[B21] Frisch M. J. Trucks G. W. Schlegel H. B. Scuseria G. E. Robb M. A. Cheeseman J. R. (2016). Gaussian 16, Revision C.01. Wallingford, CT: Gaussian, Inc.

[B22] Fuentealba P. Preuss H. Stoll H. Szentpaly V. (1982). A proper account of core-polarization with pseudopotentials: single valence-electron alkali compounds. Chem. Phys. Lett. 89, 418–422. 10.1016/0009-2614(82)80047-8

[B23] Jensen A. G. Snow T. P. (2007). New insights on interstellar gas‐phase iron. Astrophys. J. 669, 378–400. 10.1086/521638

[B24] Johnson C. E. Deegbey M. Ilic A. Kaul N. Prakash O. Wärnmark K. (2025). Shining light on the ferrous analogue: excited state dynamics of an Fe (II) hexa-carbene scorpionate complex. Dalton Trans. 54, 3586–3590. 10.1039/D5DT00139K 39936935

[B25] Jones A. P. (1990). Iron or iron oxide grains in the interstellar medium. Mon. Not. R. Astron. Soc. 245, 331–334. Available online at: https://adsabs.harvard.edu/full/1990MNRAS.245.331J .

[B26] Juanes M. Jin S. Saragi R. T. van der Linde C. Ebenbichler A. Przybilla N. (2024). Iron complexes as potential carriers of diffuse interstellar bands: the photodissociation spectrum of Fe^+^(H_2_O) at optical wavelengths. J. Phys. Chem. A 128, 1306–1312. 10.1021/acs.jpca.4c00148 38347749 PMC10895653

[B27] Kawaguchi K. Kaifu N. Ohishi M. Ishikawa S.-I. Hirahara Y. Yamamoto S. (1991). Observations of cumulene carbenes, H_2_CCCC and H_2_CCC, in TMC-1. Publ. Astron. Soc. Jpn. 43, 607–619. 10.1093/pasj/43.4.607

[B28] Koelemay L. A. Ziurys L. M. (2023). Elusive iron: detection of the FeC radical (X^3^Δ_i_) in the envelope of IRC+10216. Astrophys. J. Lett. 958, L6. 10.3847/2041-8213/ad0899

[B29] Langer W. D. Velusamy T. Kuiper T. B. H. Peng R. McCarthy M. C. Kov´acs A. (1997). First astronomical detection of the cumulene carbon chain molecule H_2_C_6_ in TMC-1. Astrophys. J. 480, 63–66. 10.1086/310622 11541460

[B30] Larsson M. Geppert W. D. Nyman G. (2012). Ion chemistry in space. Rep. Prog. Phys. 75, 066901. 10.1088/0034-4885/75/6/066901 22790651

[B31] Listyarini R. V. Gesto D. S. Paiva P. Ramos M. J. Fernandes P. A. (2019). Benchmark of density functionals for the calculation of the redox potential of Fe^3+^/Fe^2+^ within protein coordination shells. Front. Chem. doi, 391. 10.3389/fchem.2019.00391 31231631 PMC6560050

[B32] Lu T. Chen F. (2012). Multiwfn: a multifunctional wavefunction analyzer. J. Comput. Chem. 33, 580–592. 10.1002/jcc.22885 22162017

[B33] Massalkhi S. Jiménez-Serra I. Martín-Pintado J. Rivilla V. M. Colzi L. Zeng S. (2023). The first detection of SiC_2_ in the interstellar medium. Astron. Astrophys. 678, 1–8. 10.1051/0004-6361/202346822

[B34] McDonald I. Sloan G. C. Zijlstra A. A. Matsunaga N. Matsuura M. Kraemer K. E. (2010). Rusty old stars: a source of the missing interstellar iron? Astrophys. J. Lett. 717, L92–L97. 10.1088/2041-8205/717/2/L92

[B35] McGuire B. A. Asvany O. Brünken S. Schlemmer S. (2020). Laboratory spectroscopy techniques to enable observations of interstellar ion chemistry. Nat. Rev. Phys. 2, 402–410. 10.1038/s42254-020-0198-0

[B36] McKellar A. (1940). Evidence for the molecular origin of some hitherto unidentified interstellar lines. Publ. Astron. Soc. Pac. 52, 187. 10.1086/125159

[B37] Minenkov Y. Singstad Å. Occhipinti G. Jensen V. R. (2012). The accuracy of DFT-optimized geometries of functional transition metal compounds: a validation study of catalysts for olefin metathesis and other reactions in the homogeneous phase. Dalton Trans. 41, 5526–5541. 10.1039/c2dt12232d 22430848

[B38] Müller H. S. P. Schlöder F. Stutzki J. Winnewisser G. (2005). The Cologne database for molecular spectroscopy, CDMS: a useful tool for astronomers and spectroscopists. J. Mol. Struct. 742, 215–227. 10.1016/j.molstruc.2005.01.027

[B39] Murat C. Altinay G. Geoff Austein-Miller R. B. M. (2010). Vibrational spectroscopy and theory of Fe+(CH_4_)_n_ (n = 1−4). J. Phys. Chem. A 114, 11322–11329. 10.1021/jp104602k 20669921

[B40] Nash B. K. Rao B. K. Jena P. (1996). Equilibrium structure and bonding of small iron-carbon clusters. J. Chem. Phys. 105, 11020–11023. 10.1063/1.472901

[B41] Ohishi M. Kaifu N. Kawaguchi K. Murakami A. Saito S. Yamamoto S. (1989). Detection of a new circumstellar carbon chain molecule C_4_Si. Astrophys. J. Lett. 345, 83. 10.1086/185558

[B42] Pardo J. R. Cabezas C. Fonfría J. P. Agúndez M. Tercero B. De Vicente P. (2021). Magnesium radicals MgC_5_N and MgC_6_H in IRC+10216. Astron. Astrophys. 652, 5–9. 10.1051/0004-6361/202141671

[B43] Reed A. E. Weinstock R. B. Weinhold F. (1985). Natural population analysis. J. Chem. Phys. 83, 735–746. 10.1063/1.449486

[B44] Romanescu C. Galeev T. R. Sergeeva A. P. Li W. L. Wang L. S. Boldyrev A. I. (2012). Experimental and computational evidence of octa- and nona-coordinated planar iron-doped boron clusters: FeB_8_ ^-^ and FeB_9_ ^-^ . J. Organomet. Chem. 721–722, 148–154. 10.1016/j.jorganchem.2012.07.050 721

[B45] Ryzhkov M. V. Delley B. (2012). Geometry, electronic structure, and magnetic ordering of iron-carbon nanoparticles. Theor. Chem. Acc. 131, 1–18. 10.1007/s00214-012-1144-8

[B46] Shajan S. Thirumoorthy K. (2024). FeC_4_H_2_: a potential astrophysical molecule featuring planar tetracoordinate iron to unveil the mystery of missing iron in interstellar medium. ACS Earth Space Chem. 8, 2078–2089. 10.1021/acsearthspacechem.4c00178

[B47] Shajan S. Thirunavukkarsu K. Chandrasekaran V. Thimmakondu V. S. Thirumoorthy K. (2024). FeC_4_H_2_ ^2+^ encompassing planar tetracoordinate iron: structure and bonding patterns. Atoms 12, 11. 10.3390/atoms12020011

[B48] Steglich M. Chen X. Johnson A. Maier J. P. (2014). UV spectra of iron-doped carbon clusters FeC_n_ n=3–6. Int. J. Mass Spectrom. 365–366, 351–355. 10.1016/j.ijms.2014.02.006 365

[B49] Swings P. Rosenfeld L. (1937). Considerations regarding interstellar molecules. Astrophys. J. 86, 483. 10.1086/143880

[B50] Tarakeshwar P. Buseck P. R. Timmes F. X. (2019). On the structure, magnetic properties, and infrared spectra of iron pseudocarbynes in the interstellar medium. Astrophys. J. 879, 2. 10.3847/1538-4357/ab22b7

[B51] Thimmakondu V. S. Sinjari A. Inostroza D. Vairaprakash P. Thirumoorthy K. Roy S. (2022). Why an integrated approach between search algorithms and chemical intuition is necessary? Phys. Chem. Chem. Phys. 24, 11680–11686. 10.1039/D2CP00315E 35506427

[B52] Veldkamp A. Frenking G. (1992). Surprisingly high accuracy of ECP methods for predicting Fe-C bond dissociation energies of FeCH_3_ ^+^, FeCH_2_ ^+^ and FeCH^+^ . J. Chem. Soc. Chem. Commun., 118–120. 10.1039/C39920000118

[B53] Zack L. N. Halfen D. T. Ziurys L. M. (2011). Detection of FeCN (X^4^Δ_i_) in IRC+10216: a new interstellar molecule. Astrophys. J. Lett. 733, L36. 10.1088/2041-8205/733/2/L36

[B54] Zhu W. Li G. (2009). Structures and properties of small iron-doped carbon clusters. Int. J. Mass Spectrom. 281, 63–71. 10.1016/j.ijms.2008.12.012

[B55] Ziurys L. M. Apponi A. J. Guelin M. Cernicharo J. (1995). Detection of MgCN in IRC + 10216: a new metal-bearing free radical. Astrophys. J. 445, L47. 10.1086/187886

