# Supplementary material for: Computational investigation of spin-resolved energy landscapes of FeC4H2 + and their astrochemical implications
Source: Front Chem. 2026 Mar 19;14:1794369. doi: 10.3389/fchem.2026.1794369 (PMC13044147; doi:10.3389/fchem.2026.1794369)
Supplement: Supplementary file 1 [file DataSheet1.docx]

***Supporting Information of***

**Computational Investigation of Spin-Resolved Energy Landscapes of FeC_4_H_2_^+^ and Their Astrochemical Implications**

Shilpa Shajan^1^, Krishnan Thirumoorthy^1,2^*

^1^ Department of Chemistry, School of Advanced Sciences, Vellore Institute of Technology, Vellore 632014, India.

^2^ School of Computer Science and Engineering, Vellore Institute of Technology, Vellore 632014, India.

*** Correspondence:**Krishnan Thirumoorthy
[thirumoorthy.krishnan@vit.ac.in](mailto:thirumoorthy.krishnan@vit.ac.in), [kthirumoorthy@gmail.com](mailto:kthirumoorthy@gmail.com)

**
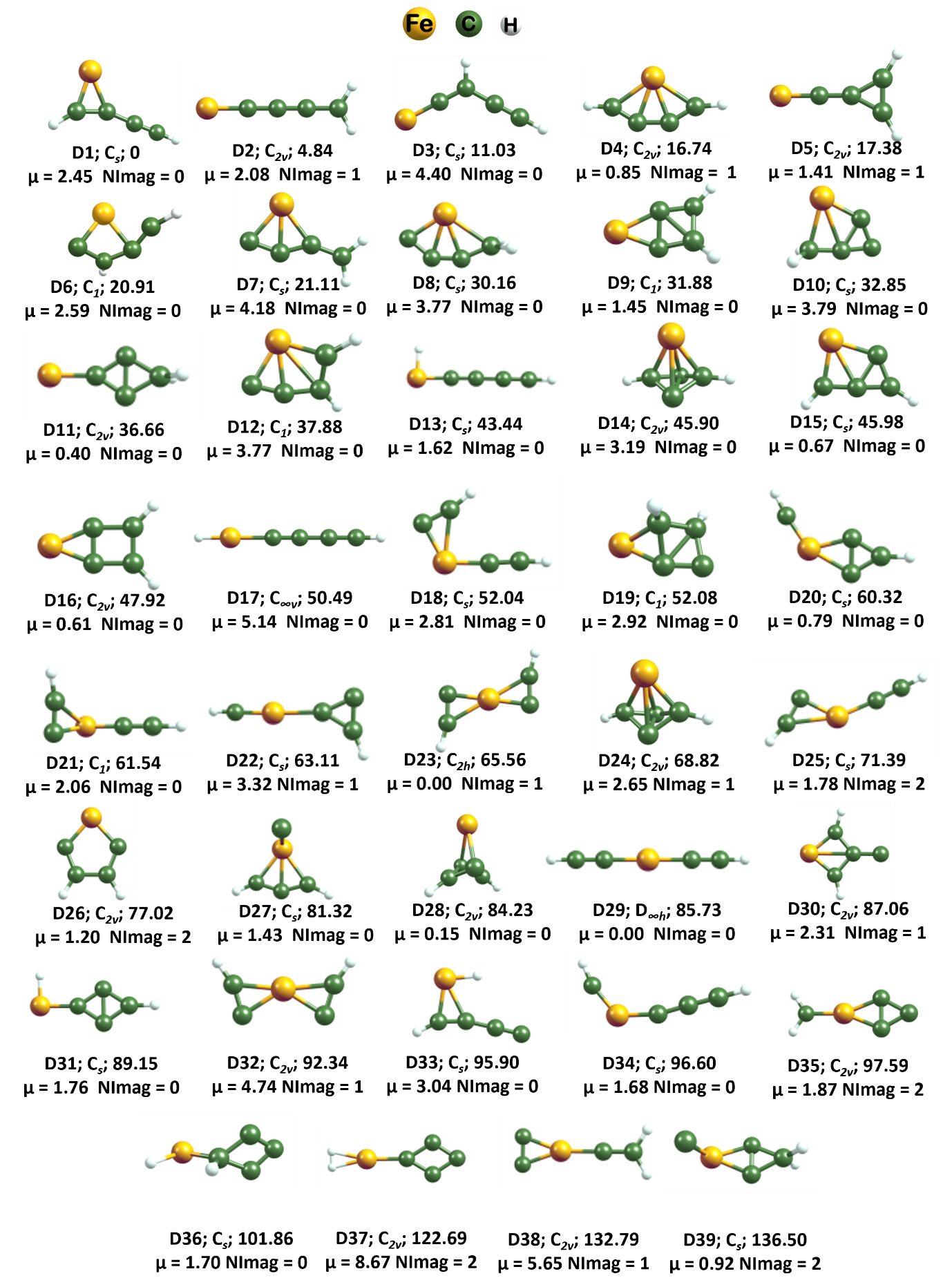
**

**Figure S1**. Optimized geometries of FeC_4_H_2_^+^ in their doublet state at U*ω*B97XD/SDD & 6-311++G(2d,2p) level. The point group, zero-point corrected relative energies (kcal mol^-1^), dipole moment (in Debye), and number of imaginary frequencies (NImag) calculated at the U*ω*B97X-D/SDD & 6-311++G(2d,2p) level.


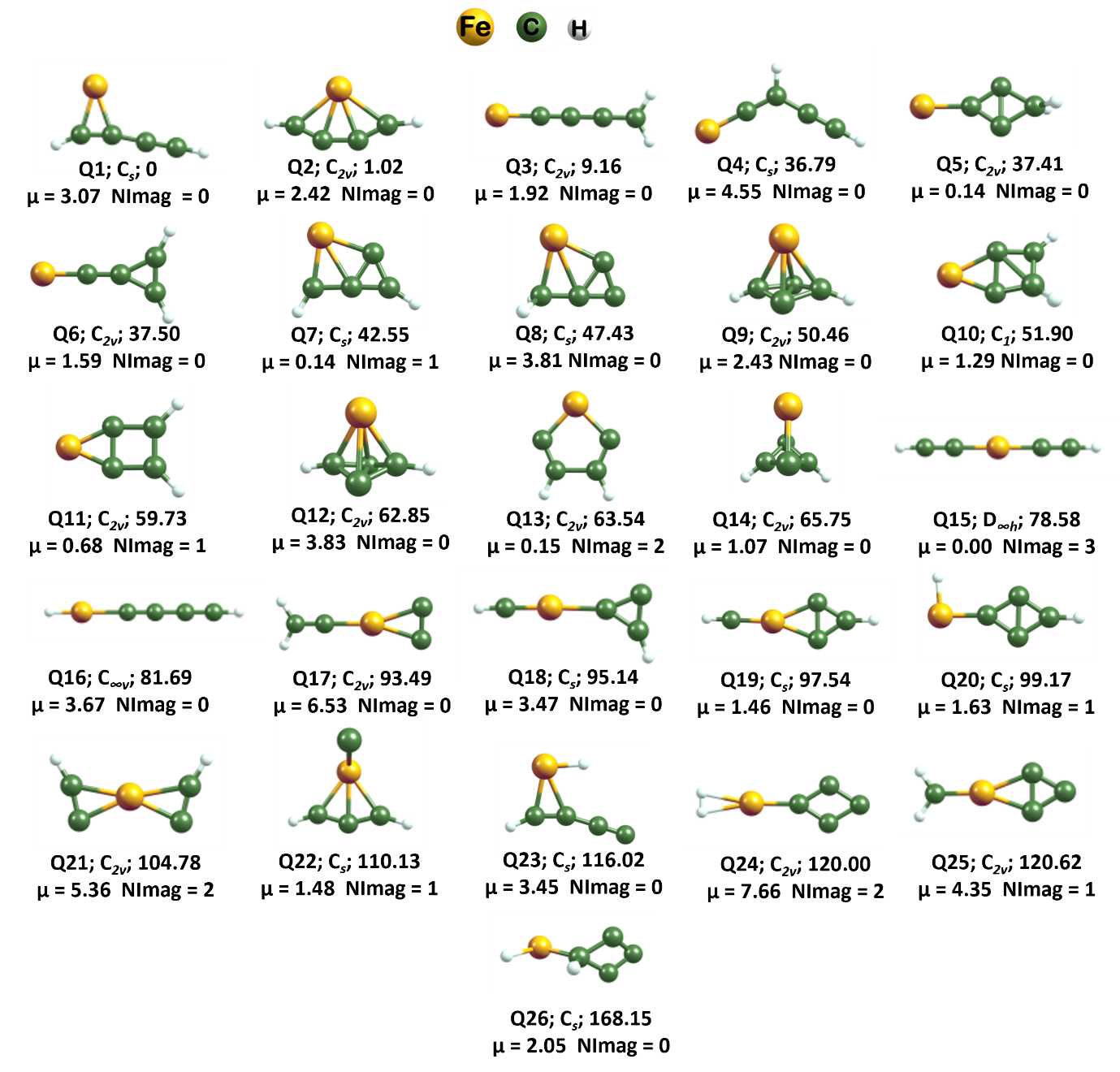


**Figure S2**. Optimized geometries of FeC_4_H_2_^+^ in their quartet state at U*ω*B97XD/SDD & 6-311++G(2d,2p) level. The point group, zero-point corrected relative energies (kcal mol^-1^), dipole moment (in Debye), and number of imaginary frequencies (NImag) calculated at the U*ω*B97X-D/SDD & 6-311++G(2d,2p) level.

**
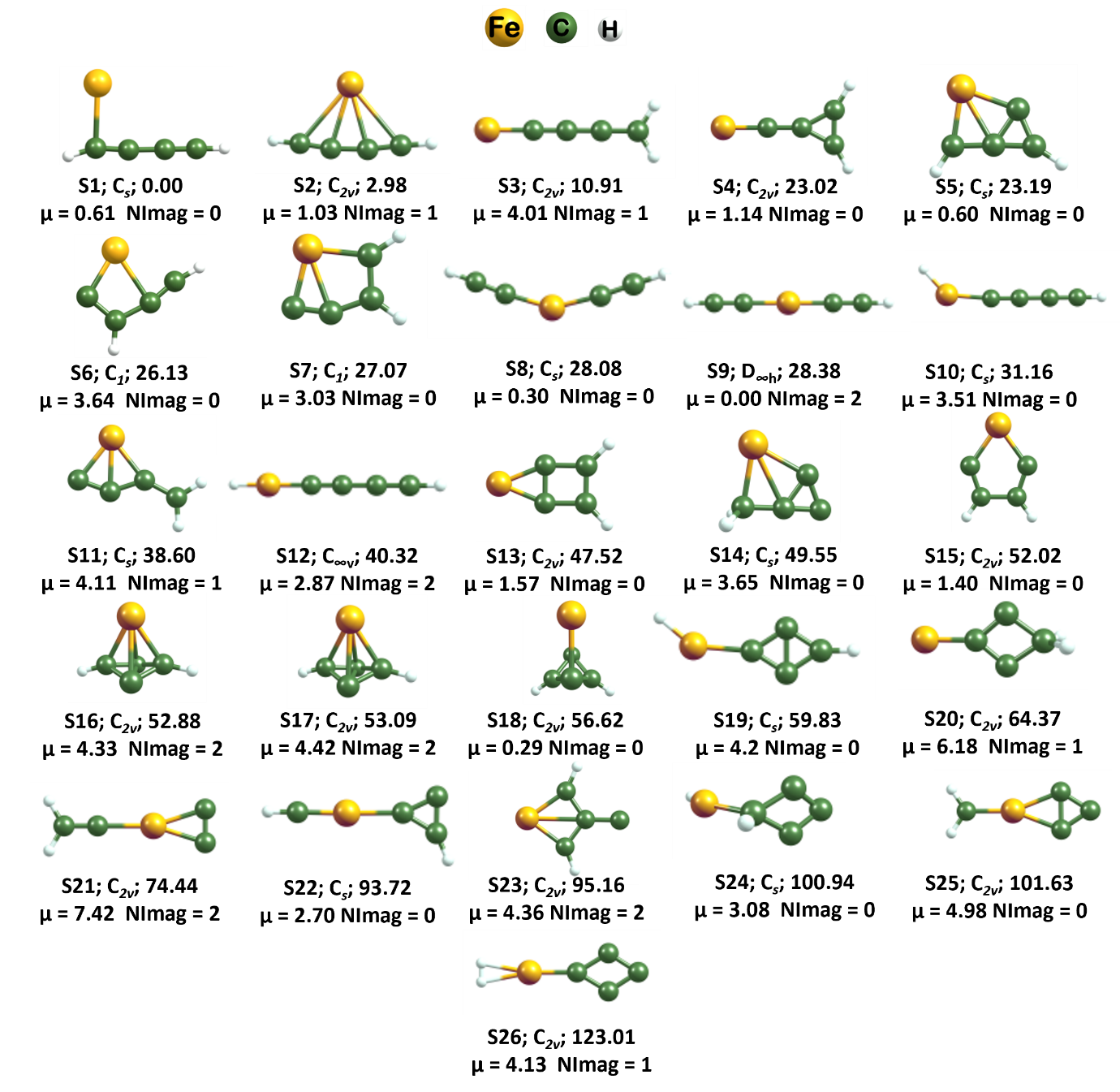
**

**Figure S3**. Optimized geometries of FeC_4_H_2_^+^ in their sextet state at U*ω*B97XD/SDD & 6-311++G(2d,2p) level. The point group, zero-point corrected relative energies (kcal mol^-1^), dipole moment (in Debye), and number of imaginary frequencies (NImag) calculated at the U*ω*B97X-D/SDD & 6-311++G(2d,2p) level.

**Table S1.** Total energy (in a.u), dipole moment (Debye), point groups, number of imaginary frequencies (NImag), zero-point correction (in a.u), ZPVE corrected total energy (E+ZPVE; in a.u), and relative energy (ΔE + ZPVE; in kcal mol^-1^), of FeC_4_H_2_^+^ in its corresponding doublet electronic state obtained at U*ω*B97XD/SDD & 6-311++G(2d,2p) level.

| **Isomer** | **Energy**  **(a.u)** | **\|µ\|**  **Debye** | **Point Group** | **NImag** | **Zero correction**  **(a.u)** | **E+ZPVE**  **(a.u)** | **∆E+ZPVE**  **(kcal/mol)** |
| --- | --- | --- | --- | --- | --- | --- | --- |
| **D1** | -277.0363032 | 2.4545 | C*_s_* | 0 | 0.038737 | -276.997566 | 0.00 |
| **D2** | -277.02642 | 2.0792 | C*_2v_* | 1 | 0.036568 | -276.989849 | 4.84 |
| **D3** | -277.0183851 | 4.4002 | C*_s_* | 0 | 0.038405 | -276.979981 | 11.03 |
| **D4** | -277.0094524 | 1.4098 | C*_2v_* | 1 | 0.038571 | -276.970882 | 16.74 |
| **D5** | -277.0097413 | 0.9764 | C*_2v_* | 0 | 0.039864 | -276.969877 | 17.38 |
| **D6** | -277.0025968 | 2.5922 | C*_1_* | 0 | 0.03835 | -276.964247 | 20.91 |
| **D7** | -277.001602 | 4.1778 | C*_s_* | 0 | 0.037684 | -276.963918 | 21.11 |
| **D8** | -276.9872756 | 3.7712 | C*_s_* | 0 | 0.037765 | -276.94951 | 30.16 |
| **D9** | -276.9850301 | 1.4475 | C*_1_* | 0 | 0.038271 | -276.946759 | 31.88 |
| **D10** | -276.9834719 | 3.7901 | C*_s_* | 0 | 0.038254 | -276.945218 | 32.85 |
| **D11** | -276.9794413 | 0.4016 | C*_2v_* | 0 | 0.04029 | -276.939151 | 36.66 |
| **D12** | -276.9753159 | 3.7772 | C*_1_* | 0 | 0.038109 | -276.937207 | 37.88 |
| **D13** | -276.9625762 | 1.6180 | C*_s_* | 0 | 0.034238 | -276.928338 | 43.44 |
| **D14** | -276.9628337 | 3.1894 | C*_2v_* | 0 | 0.038416 | -276.924418 | 45.90 |
| **D15** | -276.9615531 | 0.6681 | C*_s_* | 0 | 0.037254 | -276.924299 | 45.98 |
| **D16** | -276.9609879 | 0.609 | C*_2v_* | 0 | 0.039782 | -276.921206 | 47.92 |
| **D17** | -276.9518836 | 5.1425 | C*_∞v_* | 0 | 0.034777 | -276.917107 | 50.49 |
| **D18** | -276.9504408 | 2.8072 | C*_s_* | 0 | 0.035801 | -276.91464 | 52.04 |
| **D19** | -276.9528621 | 2.9173 | C*_1_* | 0 | 0.03829 | -276.914572 | 52.08 |
| **D20** | -276.9375143 | 0.7920 | C*_s_* | 0 | 0.036076 | -276.901439 | 60.32 |
| **D21** | -276.9353668 | 2.0565 | C*_1_* | 0 | 0.035864 | -276.899503 | 61.54 |
| **D22** | -276.9316509 | 3.3236 | C*_s_* | 1 | 0.034665 | -276.896986 | 63.11 |
| **D23** | -276.928402 | 0.0000 | C*_2h_* | 1 | 0.03531 | -276.893092 | 65.56 |
| **D24** | -276.9272656 | 2.6494 | C*_2v_* | 1 | 0.039371 | -276.887894 | 68.82 |
| **D25** | -276.9193658 | 1.7759 | C*_s_* | 2 | 0.035568 | -276.883798 | 71.39 |
| **D26** | -276.9102577 | 1.1970 | C*_2v_* | 2 | 0.035439 | -276.874819 | 77.02 |
| **D27** | -276.9028633 | 1.4266 | C*_s_* | 0 | 0.034883 | -276.86798 | 81.32 |
| **D28** | -276.9004852 | 0.1509 | C*_2v_* | 0 | 0.037156 | -276.863329 | 84.23 |
| **D29** | -276.8971132 | 0.0001 | D*_∞h_* | 0 | 0.036164 | -276.86095 | 85.73 |
| **D30** | -276.8951207 | 2.3161 | C*_2v_* | 1 | 0.03629 | -276.858831 | 87.06 |
| **D31** | -276.8880525 | 1.7556 | C*_s_* | 0 | 0.032554 | -276.855498 | 89.15 |
| **D32** | -276.8832492 | 4.7433 | C*_2v_* | 1 | 0.032835 | -276.850414 | 92.34 |
| **D33** | -276.8787593 | 3.0427 | C*_s_* | 0 | 0.034027 | -276.844733 | 95.90 |
| **D34** | -276.8789454 | 1.6844 | C*_s_* | 0 | 0.035317 | -276.843628 | 96.60 |
| **D35** | -276.8765766 | 1.8680 | C*_2v_* | 2 | 0.034537 | -276.84204 | 97.59 |
| **D36** | -276.8685886 | 1.7044 | C*_s_* | 0 | 0.033349 | -276.83524 | 101.86 |
| **D37** | -276.832539 | 8.6696 | C*_2v_* | 2 | 0.030484 | -276.802055 | 122.69 |
| **D38** | -276.8185999 | 5.6510 | C*_2v_* | 1 | 0.032652 | -276.785947 | 132.79 |
| **D39** | -276.8133089 | 0.9248 | C*_s_* | 2 | 0.033265 | -276.780044 | 136.50 |

**Table S2.** Total energy (in a.u), dipole moment (Debye), point groups, number of imaginary frequencies (NImag), zero-point correction (in a.u), ZPVE corrected total energy (E+ZPVE; in a.u), and relative energy (ΔE + ZPVE; in kcal mol^-1^), of FeC_4_H_2_^+^ in its corresponding quartet electronic state obtained at U*ω*B97XD/SDD & 6-311++G(2d,2p) level.

| **Isomer** | **Energy**  **(a.u)** | **\|µ\| Debye** | **Point Group** | **NImag** | **Zero correction**  **(a.u)** | **E+ZPVE**  **(a.u)** | **∆E+ZPVE**  **(kcal/mol)** |
| --- | --- | --- | --- | --- | --- | --- | --- |
| **Q1** | -277.10154 | 3.0694 | C*_s_* | 0 | 0.038614 | -277.06292 | 0.00 |
| **Q2** | -277.09991 | 2.4183 | C*_2v_* | 0 | 0.038622 | -277.06129 | 1.02 |
| **Q3** | -277.08634 | 1.9225 | C*_2v_* | 0 | 0.038004 | -277.04833 | 9.16 |
| **Q4** | -277.04279 | 4.5495 | C*_s_* | 0 | 0.038502 | -277.00429 | 36.79 |
| **Q5** | -277.04359 | 0.1445 | C*_2v_* | 0 | 0.040276 | -277.00331 | 37.41 |
| **Q6** | -277.04198 | 1.5887 | C*_2v_* | 0 | 0.03882 | -277.00316 | 37.50 |
| **Q7** | -277.03263 | 0.1413 | C*_s_* | 1 | 0.03751 | -276.99512 | 42.55 |
| **Q8** | -277.02518 | 3.8125 | C*_s_* | 0 | 0.03784 | -276.98734 | 47.43 |
| **Q9** | -277.01942 | 2.4326 | C*_2v_* | 0 | 0.036909 | -276.98251 | 50.46 |
| **Q10** | -277.01827 | 1.2900 | C*_1_* | 0 | 0.038058 | -276.98022 | 51.90 |
| **Q11** | -277.00694 | 0.6844 | C*_2v_* | 1 | 0.039211 | -276.96773 | 59.73 |
| **Q12** | -277.00046 | 3.8309 | C*_2v_* | 0 | 0.037709 | -276.96276 | 62.85 |
| **Q13** | -276.9971 | 0.1549 | C*_2v_* | 2 | 0.035445 | -276.96166 | 63.54 |
| **Q14** | -276.99609 | 1.0721 | C*_2v_* | 0 | 0.037948 | -276.95814 | 65.75 |
| **Q15** | -276.97146 | 0.0000 | D*_∞h_* | 3 | 0.033769 | -276.93769 | 78.58 |
| **Q16** | -276.96682 | 3.6713 | C*_∞v_* | 0 | 0.034074 | -276.93274 | 81.69 |
| **Q17** | -276.94738 | 6.5342 | C*_2v_* | 0 | 0.033454 | -276.91393 | 93.49 |
| **Q18** | -276.94635 | 3.46951 | C*_s_* | 0 | 0.035047 | -276.9113 | 95.14 |
| **Q19** | -276.94286 | 1.4605 | C*_s_* | 0 | 0.035387 | -276.90748 | 97.54 |
| **Q20** | -276.93623 | 1.6336 | C*_s_* | 1 | 0.031344 | -276.90489 | 99.17 |
| **Q21** | -276.9277 | 5.3629 | C*_2v_* | 2 | 0.031759 | -276.89594 | 104.78 |
| **Q22** | -276.91994 | 1.4798 | C*_s_* | 1 | 0.03253 | -276.88741 | 110.13 |
| **Q23** | -276.91127 | 3.4478 | C*_s_* | 0 | 0.033242 | -276.87803 | 116.02 |
| **Q24** | -276.89969 | 7.6586 | C*_2v_* | 2 | 0.028001 | -276.87169 | 120.00 |
| **Q25** | -276.90545 | 4.3462 | C*_2v_* | 1 | 0.034747 | -276.8707 | 120.62 |
| **Q26** | -276.8292 | 2.0546 | C*_s_* | 0 | 0.034236 | -276.79496 | 168.15 |

**Table S3.** Total energy (in a.u), dipole moment (Debye), point groups, number of imaginary frequencies (NImag), zero-point correction (in a.u), ZPVE corrected total energy (E+ZPVE; in a.u), and relative energy (ΔE + ZPVE; in kcal mol^-1^) of FeC_4_H_2_^+^ in its corresponding sextet electronic state obtained at U*ω*B97XD/SDD & 6-311++G(2d,2p) level.

| **Isomer** | **Energy**  **(a.u)** | **\|µ\|**  **Debye** | **Point Group** | **NImag** | **Zero correction**  **(a.u)** | **E+ZPVE**  **(a.u)** | **∆E+ZPVE**  **(kcal/mol)** |
| --- | --- | --- | --- | --- | --- | --- | --- |
| **S1** | -277.06426 | 0.7208 | C*_s_* | 0 | 0.037762 | -277.026498 | 0.00 |
| **S2** | -277.06009 | 1.0275 | C*_2v_* | 1 | 0.038119 | -277.02197 | 2.98 |
| **S3** | -277.04671 | 4.0082 | C*_2v_* | 1 | 0.036808 | -277.0099 | 10.91 |
| **S4** | -277.03023 | 1.1355 | C*_2v_* | 0 | 0.038749 | -276.99148 | 23.02 |
| **S5** | -277.02958 | 0.5953 | C*_s_* | 0 | 0.03835 | -276.99123 | 23.19 |
| **S6** | -277.02477 | 3.6382 | C*_1_* | 0 | 0.038025 | -276.98675 | 26.13 |
| **S7** | -277.02456 | 3.0347 | C*_1_* | 0 | 0.039235 | -276.98532 | 27.07 |
| **S8** | -277.02001 | 0.2986 | C*_s_* | 0 | 0.036227 | -276.98379 | 28.08 |
| **S9** | -277.01944 | 0.0000 | D*_∞h_* | 2 | 0.036098 | -276.98334 | 28.38 |
| **S10** | -277.01231 | 3.5077 | C*_s_* | 0 | 0.033194 | -276.97911 | 31.16 |
| **S11** | -277.00521 | 4.1123 | C*_s_* | 1 | 0.037429 | -276.96778 | 38.61 |
| **S12** | -276.99783 | 2.8723 | C*_∞v_* | 2 | 0.032661 | -276.96517 | 40.32 |
| **S13** | -276.99359 | 1.5650 | C*_2v_* | 0 | 0.039363 | -276.95422 | 47.52 |
| **S14** | -276.98915 | 3.6536 | C*_s_* | 0 | 0.038012 | -276.95113 | 49.56 |
| **S15** | -276.98463 | 1.4046 | C*_2v_* | 0 | 0.037249 | -276.94738 | 52.02 |
| **S16** | -276.98325 | 4.3258 | C*_2v_* | 2 | 0.037188 | -276.94606 | 52.89 |
| **S17** | -276.98292 | 4.4178 | C*_2v_* | 2 | 0.037165 | -276.94575 | 53.09 |
| **S18** | -276.97784 | 0.2869 | C*_2v_* | 0 | 0.037455 | -276.94038 | 56.62 |
| **S19** | -276.96641 | 4.2029 | C*_s_* | 0 | 0.03091 | -276.9355 | 59.83 |
| **S20** | -276.96552 | 6.1770 | C*_2v_* | 1 | 0.036926 | -276.92859 | 64.38 |
| **S21** | -276.94532 | 7.4240 | C*_2v_* | 2 | 0.03204 | -276.91328 | 74.44 |
| **S22** | -276.91907 | 2.7037 | C*_s_* | 0 | 0.035113 | -276.88395 | 93.73 |
| **S23** | -276.91622 | 4.3613 | C*_2v_* | 2 | 0.034449 | -276.88177 | 95.16 |
| **S24** | -276.90531 | 3.0755 | C*_s_* | 0 | 0.032341 | -276.87297 | 100.94 |
| **S25** | -276.90657 | 4.9810 | C*_2v_* | 0 | 0.034648 | -276.87192 | 101.64 |
| **S26** | -276.86745 | 4.1346 | C*_2v_* | 1 | 0.028037 | -276.83942 | 123.01 |
